# Low– and Medium–Socioeconomic-Status Group Members’ Perceived Challenges and Solutions for Healthy Nutrition: Qualitative Focus Group Study

**DOI:** 10.2196/40123

**Published:** 2022-12-02

**Authors:** Sander Hermsen, Alex van Kraaij, Guido Camps

**Affiliations:** 1 OnePlanet Research Center Wageningen Netherlands; 2 Wageningen University and Research Wageningen Netherlands

**Keywords:** nutrition, citizen science, socioeconomic status, digital technology

## Abstract

**Background:**

Although digital tools for healthy nutrition have shown great potential, their actual impact remains variable as digital solutions often do not fit users’ needs and barriers. This is especially poignant for priority communities in society. Involving these groups in citizen science may have great benefits even beyond the increase in knowledge of the lives and experiences of these groups. However, this requires specialized skills. Participants from priority groups could benefit from an approach that offers sensitization and discussion to help them voice their needs regarding healthy nutrition and technology to support healthy eating.

**Objective:**

This study aimed to gather insights into people’s thoughts on everyday eating practices, self-regulation in healthy eating, and skill acquisition and on applying technological innovations to these domains.

**Methods:**

Participants answered 3 daily questionnaires to garner their current practices regarding habits, self-regulation, skills, and technology use surrounding healthy eating and make it easier for them to collect their thoughts and experiences (sensitization). Within a week of filling out the 3 questionnaires, participants took part in a web-based focus group discussion session. All sessions were transcribed and analyzed using a thematic qualitative approach.

**Results:**

A total of 42 people took part in 7 focus group interviews of 6 people each. The analysis showed that participants would like to receive support from technology for a broad range of aspects of nutrition, such as measuring the effect their personal nutrition has on their individual health, providing them with reliable product information, giving them practical guidance for healthy eating and snacking, and reducing the burden of registering food intake. Technology should be easy to use, reduce burdens, and be tailored to personal situations. Privacy and cost were major concerns for the participants.

**Conclusions:**

This study shows that people from low– and medium–socioeconomic-status groups have a need for specific support in tailoring their knowledge of healthy nutrition to their own situation and see technology as a means to achieve this.

## Introduction

### Background

Nutrition is an important element of health throughout our life, from the earliest stages of infancy [1] to old age [2]. Malnutrition [3], overconsumption [4], and unbalanced diet composition [5] are strongly associated with a broad range of debilitating health conditions. The proliferation of digital technology among the population in the last decade has enabled the development and use of a broad range of health-oriented software, mobile apps, and wearable products capable of supporting us in healthy behavior change [6,7]. In digital technology for health, nutrition is one of the most frequently targeted behavioral domains [7,8]. Although digital tools for healthy nutrition have shown great potential to improve health outcomes, enhance patient experience in health care, and control or reduce costs, their actual impact remains variable. One of the main reasons for this is the fact that digital solutions often offer an approach that does not fit users’ needs and barriers, putting too much effort into knowledge attainment and too little into dealing with everyday practices and habits and social and cultural aspects of nutrition [9].

The reduced efficacy of digital health innovations is especially visible in and poignant for priority populations and communities in society [10]—people with lower income, less education, and less high-status employment than more advantageous groups in society and often referred to as vulnerable or low socioeconomic status (SES) in scientific research [11-13]. These groups require priority in digital health research and practice as their quality-adjusted life expectancy is, on average, almost a decade of healthy years lower than that of high-SES groups. This prioritization is hindered by the fact that, in scientific literature, little is known about the barriers, needs, and desires surrounding nutrition for low-SES groups in daily life [14-16].

### Citizen Science Approaches

Involving priority groups in citizen science—a research approach in which citizens themselves produce reliable scientific knowledge [17]—has great benefits beyond the increase in knowledge of the lives and experiences of these groups. The underrepresentation of individuals from priority groups [18] has known consequences on the scientific and innovative outcomes of research projects, such as interventions that work well for high-SES groups but less so for priority groups [19-21], with the current level of evidence insufficient to inform policy and practice [21]. Involving priority groups improves the chances of developing products and interventions that match the needs and possibilities of these groups. Furthermore, taking part in citizen science approaches can empower low-SES group members by increasing their skills and knowledge and providing them with a platform to share their experiences [22,23].

However, making the most of citizen science takes knowledge and awareness of those phenomena that the research looks into as well as the opportunity and skills to develop research questions and designs, make observations, apply scientific reasoning, and make sense of discoveries [24]. Unfortunately, this proves difficult for most people and even more so for people in priority groups. They could benefit from an approach that empowers them to take part in citizen science projects. Such an approach should sensitize people to the subject matter to make it easier for them to collect their thoughts and experiences. Furthermore, the approach should foster discussion to help people formulate and bring across their ideas, needs, and barriers and share and compare them with others.

Such a process, in which people from priority groups take part in discussions to help them voice their needs and desires regarding digital technology for healthy nutrition, has as yet not taken place. Research into needs and barriers regarding technology for healthy living has until now only looked at specific target groups within the population, such as older adults [25] or parents with young children [26]. These studies mainly looked at prerequisites of using technology within a given use case, such as weight management; no studies asked the target groups which use cases would interest them in the first place. However, the available literature [27-36] can give us a hint of what themes we might expect to emerge when we ask people about what they would find important in technological innovations aimed at a healthy lifestyle, such as dealing with everyday eating practices—dietary tracking, weight management, mindful eating, balanced nutrition, and eating habits; self-regulation processes (eg, in snacking behavior); and support in cooking and shopping skills.

### Objectives

Therefore, this paper had the aim of gathering insights into barriers, needs, desires, and use cases that people, especially those from priority backgrounds, experience regarding technology for healthy nutrition. To do so, the study focused on people’s thoughts on applying technological innovations to everyday eating practices, self-regulation (eg, snacking), and skill acquisition. The approach presented in this study aimed to empower priority group members to contribute to the development of research agendas in this area.

## Methods

### Overview

To gather insights into the barriers, needs, and desires surrounding healthy nutrition and technology for healthy eating, we performed a 2-phase qualitative study. In the first phase, participants answered 3 daily questionnaires to garner their current practices regarding habits, self-regulation, skills, and technology use surrounding healthy eating. Furthermore, the questionnaires served as a “sensitizer” to support participants in thinking about their healthy eating desires and needs. In the second phase, participants took part in focus group discussion sessions in which they discussed their challenges, experiences, and perceived solutions regarding healthy eating. During the focus group sessions, a short presentation on technology for health at the host institute served as further sensitization to foster further discussion on the potential of technology for supporting healthy nutrition. Participants filled out the questionnaires in the week starting on Monday, November 9, 2020; all focus group sessions were held between November 12, 2020, and November 17, 2020.

### Participants

The study aimed to include people living in the Dutch province of Gelderland and able to speak and understand Dutch. Furthermore, we aimed to include as many people from low-SES groups as possible. Indicators of low SES [37] were education (general secondary education or secondary vocational level as the highest attainment), income (<€18,390 [US $18,160.30] per annum), and employment (unemployed or otherwise). This proportion of low-SES participants should be at least as high as or higher than the low-SES prevalence in the population (ie, 20%-35% of participants). A priori sample size calculations in qualitative research are subject to conceptual debate and practical uncertainty. Saturation (ie, the moment when adding more data does not lead to new insights) is often seen as a criterion for the inclusion of more participants once the analysis has started. As a rule of thumb, 20 to 40 participants are usually considered sufficient to achieve saturation [38]. Therefore, to deal with dropout, only to be expected when conducting research under pandemic restrictions [39], we aimed to include 40 participants.

Participants were recruited from a large panel of potential participants provided by a field research agency in the Netherlands. The research team invited all members of this panel who met the eligibility criteria: living in Gelderland and a maximum educational level of secondary or vocational education. Those panel members who opted to participate were then contacted by phone to explain the study procedures. Participants received an incentive of €50 (US $49.38) to spend at a Dutch web store for taking part. All participants signed an informed consent form digitally before taking part in the study.

### Data Collection

#### Questionnaires

Participants were asked to fill out a daily questionnaire for 3 consecutive days. The goal of the questionnaires was 2-fold. First, they served as a means of gathering data on participant attitudes and behaviors surrounding healthy nutrition. Second, and more importantly, they served as a “sensitizer” to trigger participants to think about their healthy eating desires and needs. The concept of “sensitizing” is derived from participatory design practices and aims to help people think about their habits and needs in preparation for creative sessions [40]. Each questionnaire had its own theme. The first questionnaire featured questions on participant demographics, regular meals, and eating habits. The second questionnaire contained questions on self-regulatory aspects of eating behavior—drinking and snacking behavior. The third and final questionnaire contained questions on skills relevant to healthy nutrition: purchasing (healthy) food, preparing food, dealing with waste and leftovers, and using technology for healthy nutrition. The 3 questionnaires are included in Multimedia Appendix 1.

The questionnaires were sent by email at 7 PM; reminder emails were sent at 9 PM in case the questionnaire had not been filled out yet. In case participants still had not filled out the questionnaire by the next morning, they received a phone call from the supporting research company to remind them. Each questionnaire took approximately 15 to 30 minutes to fill out. Questions could be answered by selecting the relevant value from a Likert scale or list of options. Each questionnaire ended with an open question in which participants could freely describe their needs regarding the theme of the day.

For reasons of concision and because the main aim of the questionnaires was to trigger thinking about nutrition and technology in the participants rather than gather insights on nutrition habits and behaviors of the participants, the results of the questionnaires about eating habits, snacking, and drinking are not reported in the main body of this paper. An overview of these results is available in Multimedia Appendix 2. Only the results on technology use for healthy nutrition are reported in the *Results* section of this paper.

#### Focus Group Sessions

Within a week of filling out the 3 questionnaires, participants took part in one of 7 focus group discussion sessions. Owing to the restrictions because of the COVID-19 pandemic, the discussion sessions took place on the web using Microsoft Teams. Before the session, participants received the invitation for the discussion session and technical instructions for joining by email. Participants provided their permission for video recording of the sessions for analysis purposes.

Each of the 7 discussion sessions had room for 5 to 7 participants and was scheduled to last 60 minutes. A researcher served as session host; furthermore, one other researcher and an assistant took part. The session host started the session with a brief general introduction and a video and sound check for every participant. Participants were free to use the “raise hand” button to ask questions or comment at any time. The session host guided the conversation by assigning speaking turns to each participant.

The interviews were semistructured; each participant answered 5 predefined questions. In between answers, researchers and other participants could ask additional questions or comment on the provided answer. The first 3 questions were as follows: “What would you still like to know about nutrition, considering the different aspects of nutrition covered in the questionnaires?” “What would you like to change when it comes to nutrition, considering the different aspects of nutrition covered in the questionnaires?” and “How could technology support you in your challenges related to nutrition, considering the different aspects of nutrition covered in the questionnaires?” After this segment, the second researcher presented 3 examples of technological solutions to nutritional challenges: a “smart” toilet seat, a “smart” wristband, and an ingestible measurement device. Participants were then given the opportunity to ask questions or provide comments afterward. Questions 4—“What new thoughts, ideas or directions for technological solutions for nutritional challenges come to mind after hearing these examples?”—and 5—“What other aspects in daily life would you like to see more technological support, besides nutrition?”—were then discussed, and the session was concluded. The session leaders gave no further prompts or hints regarding potential themes between the main questions.

### Data Processing and Analysis

#### Questionnaires

All demographic data from the questionnaires (eg, age, gender, and education of the participants) were (pseudo-)anonymized (eg, age was divided into age groups of 18-39 years, 40-54 years, 55-64 years, and ≥65 years) and read in using R (R Foundation for Statistical Computing) [41]. For each question with Likert scales, averages and ranges were calculated. Answers to open questions were coded using the method and coding scheme derived from the discussion session analysis (see the following section).

#### Focus Group Sessions

The research team manually transcribed the recordings of the sessions. They anonymized the transcript by removing personal information. All transcripts were then read into qualitative analysis software [42] and analyzed using a method based on thematic analysis with both deductive and inductive components [43] such that insights from theory and evidence guided the analysis but, at the same time, we were open to novel themes that emerged during the analysis. Following this approach [44,45], 2 researchers (AvK and SH) first performed a primary analysis using an initial coding scheme based on expected themes derived from theory and previous literature on 29% (2/7) of the session transcripts individually and then compared their codings to ascertain similar interpretations. They then applied inductive coding to identify themes and patterns in the data that were not yet covered by the coding scheme. A further iteration of the analysis then took place to ascertain the confidence in the deductive codings. The coding scheme was then modified to better reflect emergent themes, and all relevant text segments were coded again. This step was repeated until no more issues arose.

The initial coding scheme consisted of 3 main themes, all derived from literature on determinants of healthy eating. The first theme revolved around everyday practices and habitual patterns in nutrition: meal contents, meal settings, and other regular eating-related behaviors. The second theme revolved around controlling impulses such as snacking, and the third theme revolved around skills needed for purchasing, preparing, storing, and postprocessing food. For each theme, the initial coding scheme consisted of the subthemes knowledge, desires, nontechnological solutions, and technological solutions for healthy nutrition. The illustrative quotes presented in the *Results* section were abbreviated for length and clarity.

#### How Might We Statements

To translate the results of the focus group discussions into insights suitable to inform a research agenda, we made use of *How Might We* statements [46]. This method, also known as “How-Tos,” consists of rephrasing statements about challenges or desires for healthy nutrition in such a way that they support idea generation.

### Ethics Approval

This study was exempt from approval from ethical committees under Dutch and European regulations. Under Dutch regulations, as there is no burden on the participants in this type of study (solely interview-based), it requires no ethics approval by the medical ethical committee [47]. Before enrollment, all participants provided written informed consent for the collection and use of data.

## Results

### Participants

A total of 42 people took part in 7 focus group interviews of 6 people each. In total, 38% (16/42) of the participants described their gender as man, and 62% (26/42) described it as woman. Of the 42 participants, 7 (17%) were in the 18 to 39 age group, 14 (33%) were in the 40 to 54 age group, and the remaining 21 (50%) were in the 55 to 64 age group. A total of 2% (1/42) of the participants reported their BMI as underweight (<18 kg/m^2^), 43% (18/42) were at a healthy weight (<25 kg/m^2^), 40% (17/42) were overweight (25-30 kg/m^2^), and 14% (6/42) were severely overweight (>30 kg/m^2^). Almost half (20/42, 48%) of the participants met at least one criterion of low SES, 5% (2/42) met all 3 criteria (education, profession, and income), 17% (7/42) met 2 criteria (education and profession, education and income, or profession and income), and 26% (11/42) met 1 criterion (education, profession, or income). In total, 52% (22/42) of the participants met none of the criteria; see Table 1 for an overview of low SES indicators in the participants. Of the 42 participants, 11 (26%) reported living alone, 2 (5%) lived with their partner, 15 (36%) lived with their children but without a partner, and 14 (33%) lived with their partner and children. A total of 48% (20/42) of the participants indicated some sort of health issue, with stomach and gut complaints (5/42, 12%) and type 2 diabetes (2/42, 5%) being the most frequent. All participants came from the Netherlands and were Dutch.

**Table 1:** Overview of low socioeconomic status (SES) indicators (N=42).^a^

Characteristic			Participants, n (%)
**Household gross annual income**			
	*<€14,100^b^ (US $13,923.90)*	*2 (5)*	
	€*14,100 to €29,500 (US $13,923.90-$29,131.60)*	*7 (17)*	
	€29,500 to €36,000 (US $29,131.60-$35,550.40)	8 (19)	
	€36,000 to €43,500 (US $35,550.40-$42,956.80)	7 (17)	
	€43,500 to €73,000 (US $42,956.80-$72,088.40)	8 (19)	
	€73,000 to €87,100 (US $72,088.40-$86,012.30)	5 (12)	
	>€87,100 (US $86,012.30)	3 (7)	
	No answer	2 (5)	
**Education**			
	*None or primary only or language courses only*	*1 (2)*	
	*Lower and secondary vocational education*	*5 (12)*	
	Higher levels of secondary education	7 (17)	
	Old-style vocational education (<1989)	29 (69)	
**Profession**			
	Employed in government	2 (5)	
	Employed in governmental institutes	5 (12)	
	Employed in a company	16 (38)	
	Self-employed	3 (7)	
	*Housewife or househusband*	*1 (2)*	
	*Incapacitated*	*10 (24)*	
	*Unemployed, job seeking, or social assistance*	*4 (10)*	
	Other	1 (2)	

^a^Of the 42 participants, 2 (5%) met all 3 criteria (education, profession, and income), 7 (17%) met 2 criteria (education and profession, education and income, or profession and income), and 11 (26%) met 1 criterion (education, profession, or income). In total, 52% (22/42) of the participants met none of the criteria and, therefore, were categorized as medium-SES.

^b^Categories in italics are indicators of low SES.

### Questionnaires

A large majority (38/42, 90%) of the participants indicated that they knew examples of technology to help them with healthy nutrition, be they apps, websites, or wearable devices. People who knew none were evenly distributed between the low- and medium-SES participant groups (2/42, 5% each). A total of 60% (12/20) of the participants from the low-SES group indicated using at least one technology-based solution to help with aspects of healthy nutrition, as did 64% (14/22) of the participants from the medium-SES group. Participants’ familiarity with and use of different categories of technological solutions are listed in Table 2.

**Table 2:** Knowledge and use of technology for healthy nutrition in low– and medium–socioeconomic status (SES) participants. Participants could select more than 1 category; “none of the above” excluded all other categories (N=42).

	I know of, n (%)			I already use, n (%)	
	Low SES	Medium SES	Low SES		Medium SES
Apps, websites, or smart devices to measure intake	12 (29)	8 (19)	2 (5)		1 (2)
Apps, websites, or smart devices to help prevent waste	5 (12)	8 (19)	0 (0)		0 (0)
Apps, websites, or smart devices to measure calorie need and use	11 (26)	12 (29)	4 (10)		7 (17)
Apps, websites, or smart devices to help prepare meals	9 (21)	9 (21)	10 (24)		14 (33)
Apps, websites, or smart devices to help with shopping	6 (14)	3 (7)	6 (14)		4 (10)
None of the above	2 (5)	2 (5)	8 (19)		8 (19)
Low-technology solutions (eg, pen and paper to register food)	N/A^a^	N/A	5 (12)		5 (12)

^a^N/A: not applicable.

### Focus Group Sessions

#### Overview

The original coding scheme consisted of the following main themes: everyday practices and habits in healthy eating; self-regulation of snacking and drinking; and skills in purchasing, cooking, and storing food. These main themes also emerged from the qualitative analysis. In total, 3 new main themes also emerged: user experience, privacy, and cost of technological solutions for healthy nutrition. For each of the original main themes, the original subthemes—knowledge, desires, nontechnological solutions, and technological solutions—were insufficient to reveal the structure in the retrieved codes. A new categorization of subthemes emerged: *I would like to know...*, *I notice...*, *I have difficulty with...*, *I wish...*, *My solutions are...*, *Technology could help me with...*, and *Other help I could use is...*

**Figure 1:**
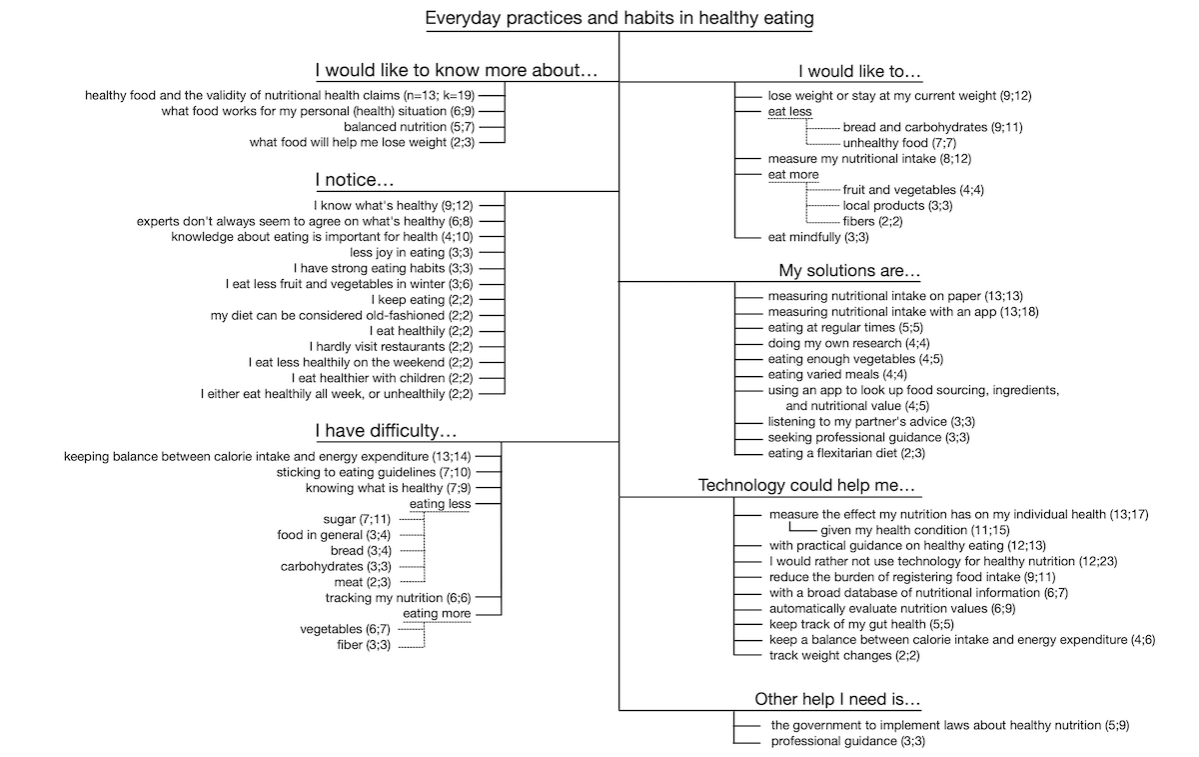
Code saturation. For each main theme and session, the occurrence of statements with codes within that theme is displayed. Largest squares: >15 mentions; medium-sized squares: 6 to 15 mentions; smallest squares: 1 to 5 mentions. k: number of unique segments about this sub-theme; n: number of unique participants mentioning this sub-theme.

Thematic saturation [48] occurred during the analysis, achieving both code saturation, with the code group structure already present after coding 29% (2/7) of the focus group interview transcripts, and meaning saturation, with all important themes developed after 71% (5/7) of the transcripts (Figure 1). Stratification of results between participants from the low- and medium-SES groups revealed that statements for every main theme occurred more or less equally between both groups, and no differences occurred.

#### Everyday Practices and Habits in Healthy Eating

**Figure 2:**
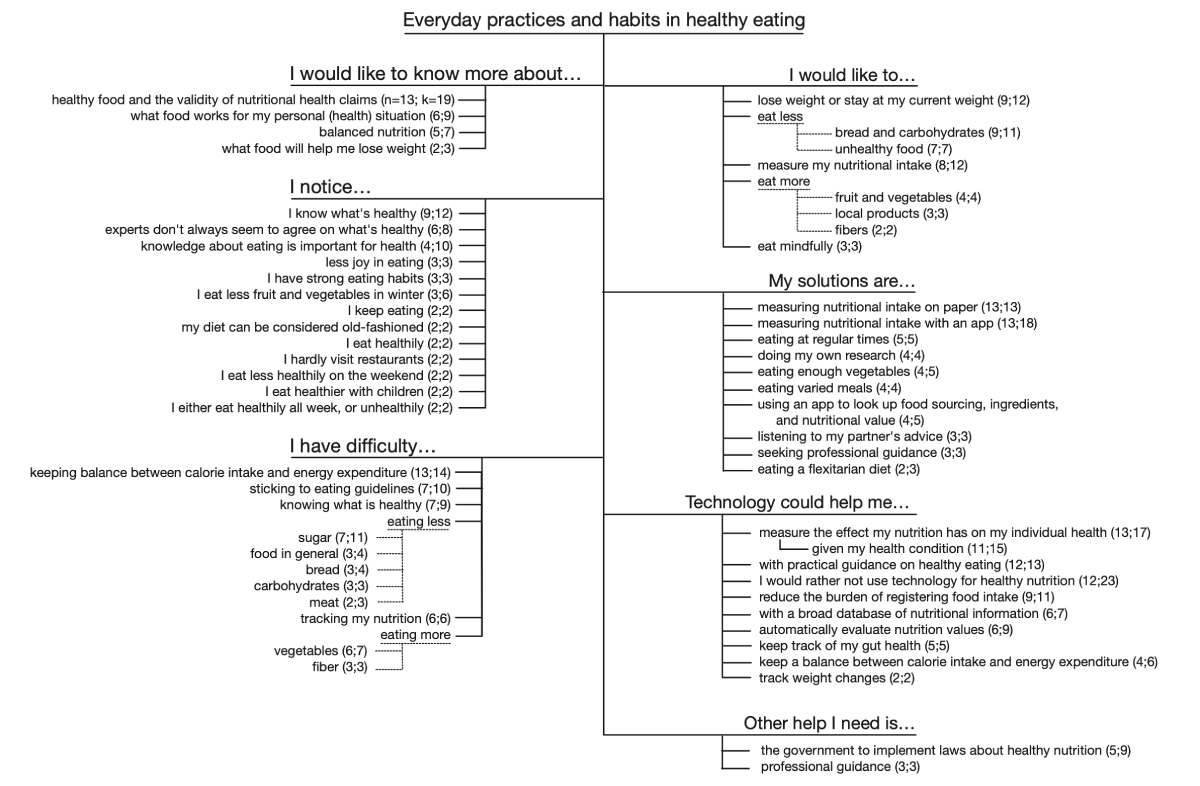
Code structure for the theme Everyday practices and habits in healthy eating. k: number of unique segments about this sub-theme; n: number of unique participants mentioning this sub-theme.

The first major theme emerging from the data concerned nutritional habits, recurring behaviors, and everyday practices in healthy eating. This theme received the most attention in the discussion sessions, with more than one-third (633/1797, 35.23%) of all the unique coded segments being within this theme. Figure 2 shows its code structure.

The analysis revealed a tension between a widespread opinion in the participants that they were well aware of what constitutes healthy nutrition on the one hand and (often at the same time) difficulties in knowing what is healthy on the other. Participant 1, for instance, stated both that they “Actually [...] feel [they] don’t really need to know much more about healthy food” and that they “could do with more information and tips and tricks on healthy eating*.*” Other subthemes within this theme appear to be related to this discrepancy—people would like to know more about the validity of health claims and notice that experts do not always seem to agree:

I have trouble getting the right information, one person says butter is good for you, the other says it’s not.

Participant 40

In science, there often seem to be competing findings. One study says that elderly people need less protein, another says you need to make sure you get enough protein. That makes it hard to do the right thing in my situation.

Participant 17

A total of 14% (6/42) of the participants expressed a wish for technology to help them determine nutritional truth by providing a broad database of nutritional information. In total, 29% (12/42) of the participants would like technology that helps them with practical guidance on healthy eating:

[a solution for] at home, when you take a piece of gingerbread, you can scan it, and the app tells you that you haven’t had your daily allowance and tells you to “go ahead, take the gingerbread, with a cup of tea.”

Participant 28

For instance, an app that I can tell “I’ve already eaten [this food] today and I’m still peckish,” and the app tells me to “just eat this, then you’ll be fine.”

Participant 10

A second major subtheme concerned personalized nutrition and knowing what effect a personal diet has on their individual health. In total, 31% (13/42) of the participants expressed a general desire for technology to evaluate the impact of nutrition on their individual health. Moreover, 26% (11/42) of the participants mentioned this in the context of their health condition. This makes technological innovations to foster personalized nutrition a desire of more than half of the participants (24/42, 57%):

I put a lot of energy into healthy nutrition, but I have reached my limits in what I can do myself, also with regard to my diabetes. I would like to know more about that.

Participant 48

I sometimes have heartburn for days at a time, and I ask myself what I shouldn’t have eaten...Too many spices? I would really like to find out why this is.

Participant 41

I have two health issues that contradict each other when it comes to nutrition. I do need help for that.

Participant 31

A related subtheme was keeping track of the balance between calorie intake and energy expenditure. A total of 31% (13/42) of the participants mentioned this as something that they struggled with, and 10% (4/42) expressed a wish to address this balance using technology:

I would like to combine the personal information of a smartwatch with food intake, that would be great. It does need to be accessible though, so the older generation [refers to self] can still deal with it.

Participant 49

To make sense of the way nutrition affects their personal situation, more than half of the participants (26/42, 62%) already tracked their food intake in one way or another. In total, 31% (13/42) of the participants used pen-and-paper solutions to do so, and another 31% (13/42) used an app. Many participants (11/42, 26%) expressed difficulties they encountered with food registration methods; when tracker apps or other pen-and-paper methods were mentioned, these mentions were often combined with the burden these solutions posed on the user (which is also a recurrent theme in the *User Experience* section):

I have tried to track [my food intake] with my activity tracker app, but that took me all day, at least it felt like that. You need to enter everything precisely and that’s just too much for me.

Participant 1

A total of 21% (9/42) of the participants mentioned a wish for technology that takes away the burden of food tracking, and another 14% (6/42) of the participants mentioned a wish for technology that automatically assesses nutritional values. In total, 29% (12/42) of the participants mentioned that they would rather not use technology at all when it comes to healthy eating but, of these 12 participants, 6 (50%) still expressed wishes for technological solutions to measure the effect of nutrition on their personal situation:

I am not an app-using person, I don’t really look up things and I don’t really care about it. [...] But I would like a watch or something that just tells me I’m getting too heavy or have been eating the wrong things.

Participant 41

Other subthemes that emerged were weight loss and maintaining a healthy weight, avoiding undesirable ingredients and foods, eating healthier food in general, and keeping track of gut health; participants would like to see technological solutions that offer practical guidance to do so. Beyond technology, 12% (5/42) of the participants would like to see extended governance in healthy eating, and 7% (3/42) would like to receive professional guidance.

#### Self-regulation

**Figure 3:**
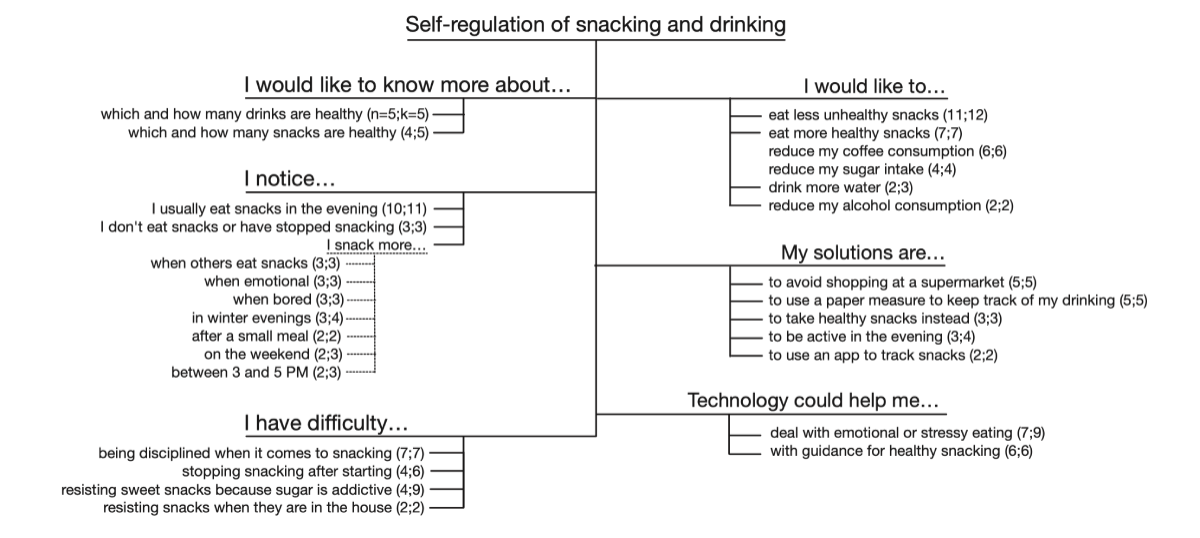
Code structure for the theme Self-regulation of snacking and drinking. k: number of unique segments about this sub-theme; n: number of unique participants mentioning this sub-theme.

The second main theme concerned self-regulation in snacking and drinking behavior, the latter mostly concerning alcohol and sweetened beverages. With 197 unique segments, this theme was less pronounced than the previous one, but the analysis revealed that this is still an issue that many people struggle with. The code structure for this theme is shown in Figure 3.

Dealing with snacking was a recurrent issue in this theme. Participants would like to know which and how many snacks could be considered healthy; noticed that there were certain times and conditions in which they snacked more; had difficulty with snacking discipline; and would like to snack less and, if they did, more healthily:

I would like to snack less when I’m bored, especially in the evenings.

Participant 30

I don’t really snack in between meals, unless I’m having a binge when I’m not feeling good about myself.

Participant 41

I consider myself an emotional eater, for instance when I am sewing, I always need something nice to take the tension away. It would help me to get notified when my stress level is getting too high.

Participant 26

Regarding technology, they would mostly like interventions to help them deal with the effect of stress and emotions on snacking (7/42, 17% of the participants) and guidance on healthy snacking (6/42, 14% of the participants):

Maybe you could develop an app that makes you happy with healthy food, without needing sugars. Because when you’re down, you tend to take chocolate and stuff, and then you notice you get tired because of the sugar. Maybe you can make some kind of mind app?

Participant 45

I know what stress does to your gut health, so I could definitely see myself using [technology] there.

Participant 49

Drinking turned out to be less relevant to the participants; they would like to know which and how many drinks could be considered healthy, drink more water, and reduce alcohol and coffee consumption. None of the participants expressed a wish to use technology to track or alter drinking behavior.

#### Skills: Shopping, Cooking, and Waste and Storage

**Figure 4:**
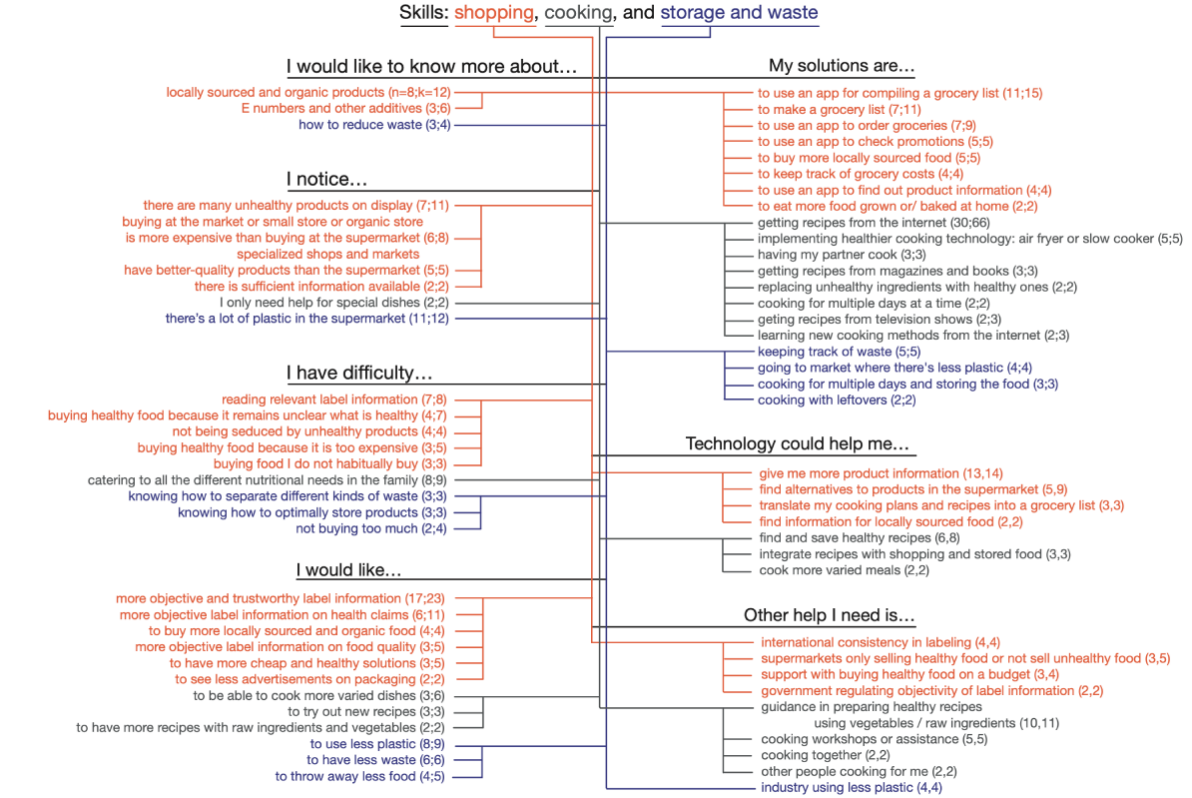
Code structure for the theme Skills: shopping, cooking, and storage and waste. k: number of unique segments about this sub-theme; n: number of unique participants mentioning this sub-theme.

The third main theme concerned the skills and information a person needs for healthy nutrition: acquiring healthy food, cooking it in a palatable manner, storing the food before preparation, and dealing with leftovers and waste. A total of 663 unique segments were included in this theme, with shopping (n=345, 52%) making up the largest part and cooking (n=201, 30.3%) and waste and storage (n=117, 17.6%) less so. The code structure for this theme is shown in Figure 4.

Regarding shopping for groceries, the largest subtheme concerned labeling information. A total of 17% (7/42) of the participants found it difficult to interpret the relevant information on the labels, and 10% (4/42) thought that what was healthy remained unclear. In total, 40% (17/42) of the participants would like to see more objective and trustworthy label information, and another 14% (6/42) would like to see more objective information on health claims:

There are so many products in stores, and the ingredients are listed in such small print, you just cannot read what is in there. [...] And there are so many different names for [for example] sugar. It’s like they are fooling you.

Participant 7

Sometimes you buy something and it says on the packaging that it’s healthy, with some sort of label too, and then you take a closer look and it turns out the food has many, many calories, and sugars, or aspartame which I’m not a fan of at all.

Participant 27

In total, 7% (3/42) of the participants particularly would like to know more about health claims regarding additives. Technology, participants thought, could help them with providing neutral product information (13/42, 31% of the participants) and find alternatives to products in the supermarket (5/42, 12% of the participants):

I always take the same, for example, potatoes, and maybe other kinds are simply better, but I don’t know that. And an app could send you a notification that I could replace the product I just took with something healthier.

Participant 48

I would like an app that can do a quick scan of the products in the store, and can give me advice on what to buy and what not, for instance because of the sugar content.

Participant 21

A second recurrent subtheme was a wish for more knowledge of (8/42, 19% of the participants) and more opportunities to buy (4/42, 10% of the participants) locally sourced and organic food. Unfortunately, even though these products are often seen as being of better quality, they are seen as more expensive. Regarding technology, 5% (2/42) of the participants would like to know more about where to find locally sourced and organic food:

I would like an app that tells me what locally sourced produce is available at this moment. So, if there’s a farmer harvesting tomatoes this week, it tells me this is a regionally sourced alternative that’s available.

Participant 42

Regarding cooking, 2 main subthemes emerged. First, many participants (8/42, 19%) expressed difficulty with catering to the different nutritional needs of family members:

I cook for different people, me and my husband, my children, and an elderly person. So, I always need to pay attention to what I cook, so everybody gets the nutrition that fits their life phase. That combination is really difficult.

Participant 28

My children do a lot of sports, and I have a sedentary profession and a tendency to snack. [...] You just can’t give everybody the same food.

Participant 45

A second subtheme was preparing varied and healthier dishes:

I mainly cook pasta now. [...] It’s just too seductive because it’s quick. I’m by myself and I don’t like cooking so I want something quick.

Participant 41

With regard to technology, most participants (30/42, 71%) used the internet as a source of recipes. Participants wanted technology to enable them to find and save healthy recipes and integrate those recipes with shopping and stored food, especially for more varied meals. This also connected to similar wishes regarding technology for shopping:

I would like an app that combines recipes with supermarket shopping lists, so you don’t have to enter everything twice.

Participant 47

[when you find a recipe] there’s an entire shopping list that you need to store somewhere and take out in the supermarket. It would be so much easier if you just can do it in one go: a cooking plan combined with a shopping list.

Participant 39

Regarding dealing with waste and storage, reducing waste, especially plastics, was the issue participants were mostly interested in (8/42, 19%). Only 2% (1/42) of the participants had a use for technology in this theme—an app that reminded them of leftovers.

#### Criteria for Use: User Experience, Privacy, and Cost

The analysis revealed 3 themes related to criteria for use: user experience, privacy, and cost. In total, this theme consisted of 198 unique segments, with 98 (49.5%) related to user experience, 46 (23.2%) concerning privacy, and 24 (12.1%) concerning financial aspects of technology for healthy nutrition.

Concerning user experience, 33% (14/42) of the participants mentioned the high burden of use that current technological solutions for healthy eating place on the user. They would welcome innovations that reduce this burden:

All those apps are way too complicated. You need to keep track of so many things for just a small result. That’s just not feasible.

Participant 28

I would look at certain apps, but it’s so much trouble to keep it up and do all the data entry that I just leave it be. I just cannot persevere.

Participant 16

I used one of those apps once, and I needed to fill in all kinds of data per day. But then I couldn’t find one [foodstuff] and then I thought, now it’s no longer accurate for me, so forget this. I never returned to the app.

Participant 30

Therefore, ease of use was mentioned by 36% (15/42) of the participants as imperative for adoption:

I want it to be without the hassle of needing to set things up; I should just have to push a button and it would tell me what I have eaten this morning and what I still need.

Participant 5

It needs to be easy, that’s right. I won’t walk away from technology, but it needs to serve me, not require me to pay constant attention and add data. It needs to support me, not the other way around.

Participant 22

In total, 14% (6/42) of the participants also wanted to ascertain that the advice they received from technology was tailored to their personal situation:

As long as I don’t get my husband’s or my son’s advice, because they are thin as planks, they can eat anything and not gain a gram. For me it’s the opposite. What if I get his advice, like, eat another eight sandwiches, that’s not going to work, is it?

Participant 49

Often [the advice] is aimed at people who do a lot of sport, not people like me who just want to move a bit more.

Participant 3

Other concerns were efficacy (4/42, 10% of the participants), reliability and validity (2/42, 5% of the participants), safety (3/42, 7% of the participants), and connectivity with other technological solutions (2/42, 5% of the participants).

In total, 55% (23/42) of the participants talked about privacy concerns with technology for healthy nutrition. Participants wanted to know who they were sharing their data with:

I don’t mind sharing things, for instance with you [researchers]. At least then the data will be beneficial for everybody. But I do want to know what is shared.

Participant 1

Even when they say they won’t use the data, I am still scared they will.

Participant 19

They wanted to have control over who obtains the data:

I want it to be designed in a way that it’s protected. That I can share the data with someone else because I allow it, for instance with your general practitioner if you have health concerns. And it needs to be safe, because in this day and age, with all the hackers and information leaks and data that gets sold on, nobody needs yet another device that will bring your entire life out in the open.

Participant 31

Participants were also well aware of the risks and negative consequences of data sharing:

What if an insurance company rejects me based on this data?

Participant 5

...you hear a lot about, for instance, a smart fridge, that others can hack into that and use it in some way or other.

Participant 19

A final recurring concern was cost. A total of 26% (11/42) of the participants mentioned high cost as a barrier to the adoption of technology for healthy nutrition:

To get comprehensive information, you often need a paid version, and that stops me.

Participant 21

I would be interested if it were covered by my health insurance. But that’s a problem in itself, because when everybody wants this technology, the insurance fees need to go up and people with limited budgets can’t afford it anymore. One way or another, it needs to be paid for.

Participant 25

I don’t have any apps because I don’t dare touch them, for fear of them costing me money.

Participant 7

Smartwatches, fitbits, yes I’ve seen them, but I just cannot afford them.

Participant 9

#### Implications for Research Agenda: How Might We Statements

To facilitate the translation of the insights from the focus group discussions into statements that could serve as starting points for future research, we reformulated the wishes participants expressed for technological support in healthy nutrition as *How Might We* statements. The full list of statements is presented in Table 3.

**Table 3:** Overview of the How Might We statements derived from participants’ expressed needs for technology support for healthy nutrition (N=42).

			Participants, n (%)
**How might we...**			
	...measure the effect individual nutrition has on a person’s unique health situation?	24 (57)	
	...provide people with detailed product information when shopping?	13 (31)	
	...give practical guidance for healthy and varied eating?	12 (29)	
	...reduce the burden of registering food intake?	9 (21)	
	...help people deal with emotional or stressy eating?	7 (17)	
	...provide a broad database of nutritional information?	6 (14)	
	...automatically evaluate nutrition values?	6 (14)	
	...give practical guidance for healthy snacking?	6 (14)	
	...help people find and save healthy recipes?	6 (14)	
	...help people keep track of their gut health?	5 (12)	
	...help people find products and alternatives for products in the supermarket?	5 (12)	
	...help people keep a balance between calorie intake and energy expenditure?	4 (10)	
	...help people translate cooking plans and recipes into grocery lists?	3 (7)	
	...help people integrate recipes with shopping and food stored at home?	3 (7)	
	...help people track weight changes?	2 (5)	
	...provide information on locally sourced food?	2 (5)	
	...help people cook more varied meals?	2 (5)	
**In such a way that...**			
	...it reduces the burden of keeping track of nutrition and reminding oneself of goals?	23 (55)	
	...the innovation is easy to use?	17 (40)	
	...it is tailored to people’s personal situation?	6 (14)	
	...is effective?	4 (10)	
	...is safe?	3 (7)	
	...is reliable and valid?	2 (5)	
	...is connected to other solutions?	2 (5)	

## Discussion

### Principal Findings

This study aimed to gather insights into use cases, barriers, and needs for technology in people from priority backgrounds to support them in healthy nutrition. To do so, participants from lower- and medium-SES groups filled out questionnaires and took part in a focus group discussion meeting. The study showed that participants would like to receive support from technology to measure the effect their personal nutrition has on their individual health; provide them with reliable product information; give them practical guidance for healthy eating and snacking; reduce the burden of registering food intake; help them deal with emotional or stress-based snacking; automatically evaluate nutritional values of their food intake; help them find and save healthy, varied recipes and translate these recipes, combined with stored food at home, into shopping lists; help them keep track of gut health; help them find alternatives to unhealthy products; help them keep a balance between calorie intake and energy expenditure; help them track weight changes; and provide information on locally sourced food. Technology should be easy to use; reduce the burden of keeping track of nutrition and intervening in healthy eating; and be tailored to their personal situation, effective, safe, reliable, valid, and connected to other “smart” solutions. Privacy and cost were major concerns for the participants.

The results show that tensions exist in the way people perceive and act upon healthy eating. On the one hand, participants indicated that they had all the knowledge they needed for healthy nutrition but, by contrast, (the same) participants told us that they were often confused by contradictory health claims and had difficulty putting their (often abstract) knowledge into everyday practice. A similar tension can be found in technology use. More than one-quarter of the participants (12/42, 29%) told us that they would rather refrain from using technology for healthy eating but, at the same time, had clear opinions on how technology could still help them. This tension could very well be driven by the experience participants have with current solutions, which are often burdensome and insufficiently tailored to their personal needs and situations. Personalized feedback and guidance was the number-one use case described by the participants, especially regarding dealing with often complex health issues and different needs in the family. However, not every barrier, concern, or need described by the participants was accompanied by a wish for a technological solution. Abundance of plastic packaging was mentioned by many participants (11/42, 26%) as a concern, but none of them felt the need for digital health innovations to deal with this issue.

The aim of this study was to reach participants from lower- and medium-SES groups. The results showed that 45% (19/42) of the participants met at least one marker of membership of these groups. None of the remaining participants were from high-status groups. This makes this study successful, but caution regarding the generalizability of the results remains necessary. First, the markers describing low SES are vague and apply to a wide range of profiles, from people without formal education but with successful careers to people who have received higher education but are currently unemployed. This vagueness in definitions is not unique to this research but applies to all studies dealing with priority populations [37]. Second, selection bias is likely to have occurred in the recruitment of the participants. To take part in the study, participants had to be members of a specific research panel and express their interest in the study. This means that our results represent the opinions and interests of a convenience sample, and people without an interest in technology are not likely to have applied for the study. However, as these people are also most likely to be early adopters of technology to support them in healthy eating, the results of this study are still relevant to inform further research and innovation even if some selection bias occurred. Third, researcher bias, in which the interests of the researchers affect question order, question content, and interview procedures, can have occurred. All authors are professionally involved in research into and the development of technological innovations, which may have influenced the research. However, as 80% of innovations fail within 2 years of launch because of limited connection to everyday practices and barriers of users [49], this involvement also entails a sincere interest in what people do not find interesting or engaging in technology or when technology fails in supporting people in changing their behavior. Overall, the study managed to engage different voices than is usually the case, with some caveats given the potential for bias. Further research can support or refute the conclusions drawn in this paper.

The questionnaire results showed that the participants in this study can be seen as typically Dutch eaters, with sandwiches for breakfast and lunch and a hot meal for dinner often consisting of potatoes, vegetables, and meat [50]. This shows that we managed to engage the “common person” in this research but, of course, it limits the extent to which these results can be generalized to other diets and cultures. In addition to nutritional content, Dutch eating culture sees nutrition as an individual matter or, at the most, a matter of the nuclear family [51]. Many (if not most) other cultures lay more emphasis on social practices of eating [51]. It is to be expected, but open to future research, that people from these cultures would place more value on solutions that cater to different needs within the (extended) family or enhance social or festive aspects of healthy eating [52-55].

The desire for personalization of healthy eating corresponds with recent evidence (see the study by König et al [28] for a review); technological interventions for healthy nutrition need to be customizable and tailored to individual needs. Usability issues [56-60], privacy concerns [58,61], and cost concerns [57,62] are also well known as barriers to adoption and sustained engagement with technological innovations.

This study used a novel method derived from participatory design. Therefore, the study can serve as an example of a case study of citizen science approaches that go beyond simply having citizens gather data for preset research questions. The approach described in this paper is a first step toward the development of a well-defined method for the first stages of participatory research [63], in which citizens have the opportunity to voice their thoughts, concerns, and desires about what is researched. In the follow-up stages, the research questions generated by the approach described in this paper can then be explored by the citizens involved in the discussion and others. This study shows the promise of this approach as it produced a broad range of research questions that could be further explored. The sensitizer phase, using questionnaires to help shape people’s thoughts about subjects such as habitual eating, self-regulation, and skills, did its work in that participants seemed well prepared to take part in the discussion. Sensitizing people to subjects could potentially carry with it a risk of social desirability bias in questionnaire and interview responses. However, this bias is mitigated by a better preparedness in the participant—they have already had the opportunity to form their own opinion on the subject and are less likely to be subject to answering biases during the session [64].

The sensitizing exercise performed during the discussion session, in which we showed the participants examples of innovations, was much less successful. After the presentation, hardly any new themes emerged. The exercise did not seem to help participants think about technology beyond what they already knew and used, as evidenced by the fact that the ideas for solutions that the participants mentioned were mostly similar to the apps and websites that they already used. A more immersive approach could be more fruitful in this case, such as “provotyping” [65,66]. In this approach, a combination of “provocation” and “prototyping,” participants receive gentle and safe “provocations” through experiencing (potential) innovations that could help them deal with the subject matter. Often, such “provotypes” lead to discussing latent cultural norms and taboos, automatic behavioral patterns such as deeply entrenched habits, and other processes and attitudes that normally escape conscious scrutiny. Ideally, people should experience the prototype for an extended period, not just as part of a discussion session.

The research delivered a range of research questions (reflected in the list of *How Might We* statements) on the barriers, needs, desires, and use cases people from priority backgrounds experience regarding technology for healthy nutrition. These statements have already proved useful to help inform the discussion on the research agenda on technology for healthy nutrition at the host institution. They helped shape conversations with potential partners from academia and industry, and this approach is now used in other scientific programs within the organization. However, the establishment of a research agenda is often complex and must align the needs and demands of practice (industry and health care), science, and funding agencies. Furthermore, a technology at an advanced readiness level must be available for further development. All this entails that, in practice, the impact of citizens’ input on research agendas will remain smaller than desired. Nonetheless, this approach can make sure that the voice of the citizen is considered, which in itself is already a great step forward.

### Conclusions

This study provided an overview of challenges, needs, and barriers that people from low- and medium-SES groups see when it comes to healthy nutrition. The results show that these people, even though they think of themselves as having knowledge of what constitutes healthy eating, are in need of specific support when it comes to knowing what is healthy for their specific situation and specific support for changing everyday eating practices and habits and obtaining skills needed for healthy eating. The study also showed how technology can play a role in supporting these people and that usability, privacy, and cost need to be considered. Finally, the study provided an approach to help people from priority groups voice their needs and concerns and can serve as a blueprint to use input from these groups to inform research and development agendas.

## Appendix

Multimedia Appendix 1 Sensitizing questionnaires.

Multimedia Appendix 2 Eating behavior questionnaire overview.

## Abbreviations

SES: socioeconomic status

## Data Availability

The data sets used and analyzed during this study are available from the corresponding author upon reasonable request.
