# Supplementary material for: Low– and Medium–Socioeconomic-Status Group Members’ Perceived Challenges and Solutions for Healthy Nutrition: Qualitative Focus Group Study
Source: JMIR Hum Factors. 2022 Dec 2;9(4):e40123. doi: 10.2196/40123 (PMC9758634; doi:10.2196/40123)
Supplement: Multimedia Appendix 1 [file humanfactors_v9i4e40123_app1.pdf]

Multimedia Appendix 1: Sensitizing Questionnaires

Day 1: This is the questionnaire that was sent at the end of the first day of the Citizen Science nutrition project.

1. How do you live? Mark only one option.
Alone
With partner
With partner and child(ren)
With child(ren)
With roommate / roommates
With parents
Otherwise:

2. How many children under 18 live in your household?
(open answer)

3. How many children under 5 live in your household?
(open answer)

4. What was your breakfast today?
(open answer)

5. What did you drink with this breakfast?
(open answer)

6. Who prepared this breakfast? You can indicate multiple answers. Check all applicable options.
yourself
your child(ren)
your partner
your parent(s)
Other:

7. approximately what time did you have breakfast?
Example: 8:30
(open answer)

8. Who did you have breakfast with? You can indicate multiple answers. Check all applicable options.
Alone
your partner
your child(ren)
your colleague(s)
your parent(s)
Others:

9. Where did you have breakfast?
(open answer)

10. What kind of waste did you have after breakfast? You can indicate multiple answers.
Check all applicable options.
none
washing up
paper
plastic
food scraps
glass
Other:

11. Did you eat this breakfast at home or elsewhere? Mark only one option.
at home
elsewhere:

12. In the case of going out, how did you arrange this breakfast?
Mark only one option.
I bring food from home
Food is being arranged for me
I buy food at the location (restaurant, canteen, etc.)
I buy food in advance (grocery store, gas station, vending machine, etc.)
Other:

13. In the case of at home, where did you eat this breakfast? Mark only one option.
At the table
On the couch
standing
During other activities
Other:

14. Is this your normal breakfast on a weekday? Mark only one option.
Yes
no
I do not know

15. If not, what was different?
(open answer)

16. What did your lunch consist of today?
(open answer)

17. What did you drink with this lunch?
(open answer)

18. Who prepared this lunch? You can indicate multiple answers. Check all applicable options.
yourself
your child(ren)
your partner
your parent(s)
Other:

19. what time did you have lunch approximately?
Example: 8:30
(open answer)

20. Who did you have lunch with? You can indicate multiple answers. Check all applicable options.
alone
your partner
your child(ren)
your colleague(s)
your parent(s)
Other:

21. Where did you have lunch?
(open answer)

22. What kind of waste did you have after lunch? You can indicate multiple answers. Check all applicable options.
none
washing up
paper
plastic
food scraps
glass
Otherwise:

23. Did you eat this lunch at home or elsewhere? Mark only one option.
at home
elsewhere:

24. In the case of elsewhere, how did you arrange this lunch?
Mark only one option.
I bring food from home
Food is being arranged for me
I buy food at the location (restaurant, canteen, etc.)
I buy food in advance (grocery store, gas station, vending machine, etc.)
Other:

25. In the case of at home, where did you eat this lunch? Mark only one option.

At the table
On the sofa
standing
During other activities
Other:

26. Is this a normal lunch for you on a weekday? Mark only one option.
Yes
no
I do not know

27. If not, what was different?
(open answer)

28. What was your dinner today?
(open answer)

29. What did you drink with this dinner?
(open answer)

30. Who prepared this dinner?
You can indicate multiple answers. Check all applicable options.
yourself
your child(ren)
your partner
your parent(s)
Others:

31. what time did you eat dinner? Example: 8:30
(open answer)

32. Who did you have dinner with?
You can indicate multiple answers. Check all applicable options.
alone
your partner
your child(ren)
your colleague(s)
your parent(s)
your roommate(s)
Others:

33. Where did you have dinner tonight?
(open answer)

34. Did you eat this dinner at home or elsewhere? Mark only one option.
at home
elsewhere:

35. In the case of elsewhere, how did you arrange this dinner?
Mark only one option.
I bring food from home
Food is being arranged for me
I buy food at the location (canteen, restaurant, etc.)
I buy food in advance (grocery store, petrol station, vending machine, etc.)
Other:

36. In the case of at home, where did you eat this dinner? Mark only one option.
At the table
On the sofa
On the sofa with the TV on
Standing
During other activities
Other:

37. What kind of waste did you have after dinner? You can indicate multiple answers. Check all applicable options.
none
washing up
paper
plastic
food scraps
glass
Otherwise:

38. Which of the following statements is most accurate for how you eat your dinner? Mark only one option.
I fill my plate completely and then usually take seconds
I fill my plate completely and that's enough
I take a smaller portion and then usually take seconds
I take a smaller portion and that's enough for me
I always try to make sure that all food that is prepared gets eaten
Other:

39. Is this a regular dinner for you? Mark only one option.
Yes
no
I do not know

40. If not, what was different?
(open answer)

41. Do you feel that you ate healthy today? Mark only one option.
Yes
no

I do not know

42. If so, what made your food healthy today?
(open answer)

43. Did you eat healthier or less healthy today than other days? Mark only one option.
Much less healthy
Less healthy
Just as healthy
Healthier
Much healthier
I don't know

44. Do you often eat vegetarian or vegan meals? Mark only one option.
I only eat vegetarian
I only eat vegan
I eat vegetarian as much as possible
I eat vegan as much as possible
I sometimes eat vegetarian
I sometimes eat vegan
I don't care about this
Other:

45. In the case of "I eat only or as much vegetarian as possible", why do you eat only / as much vegetarian as possible?
(open answer)

46. In the case of "I eat only or as much vegan as possible", why do you eat only/as much vegan as possible?
(open answer)

Below are some statements that you can answer by choosing an option from strongly disagree to strongly agree.
47. I would like to know more about how healthy my diet is. Mark only one option.
48. I would like to know more about healthy eating in general.
49. I believe that food should above all be tasty.
50. I think that food should above all be fun.
51. I am a fast eater.
52. I am an attentive eater, I eat mindfully.
53. I would like to lose weight.
54. I am currently on a diet
55. I have been on a diet in the past.
56. I sometimes have problems with my stomach or digestion.
57. I sometimes have problems with my bowel movements (constipation, diarrhoea).
58. I sometimes have little energy.
59. I sometimes sleep badly.

60. Would you like to know more about the above topics, such as your breakfast, lunch, and dinner?
(open answer)

61. If yes, what questions do you have?

62. What would you like to change about your breakfast, lunch, or dinner?

Day 2: This is the questionnaire that was sent at the end of the second day of the Citizen Science Nutrition project.

1. Did you eat one or more snacks or snacks this morning? Mark only one option.
Yes
No 2.

2. If so, what did you eat as a snack(s) or snack(s) this morning?
(open answer)

3. If so, what time did you eat this/these snack(s) or snack(s) this morning?
Possibility to indicate multiple times here. Example: 8:30
(open answer)

4. If so, who did you have a snack with this morning? You can indicate multiple answers. Check all applicable options.
Alone
With your partner
With your parent(s)
With your child(ren)
With your colleague(s)
Otherwise:

5. If so, who made the choice to grab this/these snack(s)? Mark only one option.
yourself
someone else:

6. Did you have a drink this morning? Mark only one option.
Yes
No

7. If so, what did you drink this morning?
(open answer)

8. If so, what time did you drink this morning? Possibility to indicate multiple times here. Example: 8:30, 10:15

9. If so, who did you drink with this morning? You can indicate multiple answers. Check all applicable options.
Alone
With your partner
With your parent(s)
With your child(ren)
With your colleague(s)
Others:

10. Was this morning different for you than usual in terms of snack(s) and drink(s)? Mark only one option.
Yes
No

11. If so, what was different this morning?
(open answer)

12. What is your favorite morning snack?
(open answer)

13. What is your favorite morning drink?
(open answer)

14. Did you eat one or more snacks this afternoon? Mark only one option.
Yes
No

15. If so, what did you eat as a snack(s) this afternoon?
(open answer)

16. If so, what time did you eat this/these snack(s) this afternoon? Possibility to indicate multiple times. Example: 8:30, 10:15
(open answer)

17. If so, with whom did you eat this/these snack(s) this afternoon? You can indicate multiple answers. Check all applicable options.
alone
with your partner
with your parent(s)
with your child(ren)
with your colleague(s)
Other:

18. If so, who made the choice to eat this/these snack(s)? Mark only one option.
Myself
Someone else:

19. Did you have a drink this afternoon? Mark only one option.
Yes
No

20. If so, what did you drink this afternoon?
(open answer)

21. If so, what time did you drink this afternoon? Possibility to indicate multiple times.
Example: 8:30, 10:15

(open answer)

22. If so, who did you drink with this afternoon? You can indicate multiple answers. Check all applicable options.
Alone
With your partner
With your parent(s)
With your child(ren)
With your colleague(s)
Others:

23. Was this afternoon different for you than usual in terms of snack(s) and drink(s)? Mark only one option.
Yes
No

24. If so, what was different this afternoon?
(open answer)

25. What is your favorite afternoon snack?
(open answer)

26. What is your favorite afternoon drink?
(open answer)

27. Did you eat a snack(s) tonight? Mark only one option.
Yes
No

28. If so, what did you eat as a snack(s) tonight?
(open answer)

29. If so, what time did you eat this/these snack(s) tonight?
Possibility to indicate multiple times. Example: 8:30, 10:15
(open answer)

30. If so, with whom did you have this/these snack(s) tonight? You can indicate multiple answers. Check all applicable options.
Alone
With your partner
With your parent(s)
With your child(ren)
With your colleague(s)
Others:

30. If so, who made the choice to eat this/these snack(s) tonight? Mark only one option.
Myself

Someone else:

31. Did you have a drink tonight? Mark only one option.
Yes
No
32. If so, what did you drink tonight?
(open answer)

33. If so, what time did you drink tonight?
Possibility to indicate multiple times. Example: 8:30, 10:15
(open answer)

35. If so, who did you drink with tonight? You can indicate multiple answers. Check all applicable options.
Alone
With your partner
With your parent(s)
With your child(ren)
With your colleague(s)
Others:

36: Was this evening different for you than usual in terms of snack(s) and drink(s)? Mark only one option.
Yes
No

37. If so, what was different this evening?
(open answer)

38. What is your favorite evening snack?
(open answer)

39. What is your favorite drink in the evening?
(open answer)

40. Are you a fast eater or a slow eater? Mark only one option.
Fast
Slow
I don't know

41. Do you ever take extra supplements or vitamins? Mark only one option.
Yes
no
I do not know

42. If so, what supplements or vitamins do you take?
(open answer)

43. If so, why are you taking these supplements or vitamins?
(open answer)

44. How do you know if you are a fast eater or slow eater?
(open answer)

45. Do you ever get up at night to eat something? Mark only one option.
Yes
No

46. Did you get out of bed last night to get something to eat?
Yes
No

47. How much alcohol do you drink per week? 1 drink is 1 glass (1 dl) wine, 1 glass (3 dl) beer or 2 cl spirits. Mark only one option.
None or less than 1 drink per week
1 to 3 drinks per week
4-7 drinks a week
8-12 drinks a week
more than 12 drinks per week

46. Who do you drink alcohol with (at least once a week)? You can indicate multiple answers. Check all applicable options.
Alone
With my partner
With my friends
With my colleagues
Others:

47. How many soft drinks do you drink per week?
1 glass (~16 cl) counts as 1 drink. 1 can (33 cl) counts as 2 drinks. 1 bottle (0.5 L) counts as 3 drinks. Mark only one option.
No or less than 1 drink per week
1 to 3 drinks per week
4-7 drinks a week
8-12 drinks a week
12-16 drinks a week
16-20 drinks a week
20-25 drinks a week
25-30 drinks a week
More than 30 drinks a week

48. How much coffee do you drink per day? Mark only one option.
None or less than 1 coffee per day
1 to 3 coffees per day

4-7 coffees a day
more than 7 coffees a day

51. Do you ever feel that you are hungry? Mark only one option.
never
sometimes
often
usually
always

52. Do you notice when you are full? Mark only one option.
I notice that I am full and then stop eating
I notice I'm full but eat more
I sometimes don't notice that I'm full and sometimes I eat too much
I hardly ever notice that I am full and often eat too much
I hardly ever notice that I am full and therefore clean my plate and then eat no more
Other:

52. Do you ever eat when you are stressed? Mark only one option.
never
sometimes
often
usually
always

53. Do you ever eat when you are unhappy? Mark only one option.
never
sometimes
often
usually
always

54. Do you ever eat when you are angry? Mark only one option.
never
sometimes
often
usually
always

55. Do you ever eat when you are bored? Mark only one option.
never
sometimes
often
usually
always

57. Would you like to know more about the above topics, such as your snacks and drinks?
Mark only one option.
Yes
no
I do not know

58. If so, what questions do you have about this?
(open answer)

59. What would you like to change about your snacking and drinking behaviour?
(open answer)

Day 3: This is the questionnaire that was sent out at the end of the third day of the Citizen Science Nutrition project.

1. Who does the shopping in your household? Mark only one option.
I always do the shopping
I usually do the shopping, sometimes someone else (partner, roommate, child, parent)
I do the shopping about as often as others in my household (partner, roommate, child, parent) Others (partner, roommate, child, parent) usually do the shopping, I sometimes do that
Others (partner, roommate, child, parent) always do the shopping, I never do that
Otherwise:

2. If you answered to the previous question that others also (sometimes) do the shopping in your household, who does the shopping in your household?

3. How often do you shop? Mark only one option.
Once a month or less
2-3 times a month
1-2 times a week
3-4 times a week
more than 4 times a week

4. Who decides what is bought when shopping? You can indicate multiple answers. Check all applicable options.
yourself
Your partner
Your child(ren)
Your parent(s)
Your roommates
Whoever does the shopping at that moment
Otherwise:

5. When shopping, how important is the price of a product to you? Mark only one option.
Scale of 1–5: Very unimportant to Very important

6. When shopping, how important is how healthy a product is to you? Mark only one option.
Scale of 1–5: Very unimportant to Very important

7. When shopping, how important is how tasty a product is to you? Mark only one option.
Scale of 1–5: Very unimportant to Very important

8. When shopping, how important is it to you how good a product is for the environment?
Scale of 1–5: Very unimportant to Very important

9. True or not? I find shopping quite difficult. Mark only one option.
Scale of 1–5: Not true at all to Certainly true

10. Do you usually buy the same things? My shopping is... Mark just one option.
Scale of 1–5: Always very different to Always exactly the same

11. Would you like some help with the shopping? Mark only one option.
Yes
no
I do not know

12. If so, what kind of help would you like?
(open answer)

13. Do you ever use an app, website, or smart device to help you with your shopping? Mark only one option.
Yes
no
I do not know

14. If so, what do you use that technology for?
(open answer)

15. Do you wish there was better technology (app, website, smart devices) to help you with your shopping? Mark only one option.
Yes
no
I do not know

16. What would that technology do for you?
(open answer)

17. Have you cooked yourself this week? Mark only one option.
Yes, practically every meal
Yes, most of the meals
Yes, some meals
Yes, once
No, I didn't cook myself

18. Who else cooks in your household?
(open answer)

19. In answer to previous question, how often do they cook?
(open answer)

20. Do you often cook the same? Mark only one option.
Scale 1–5: Almost always the same to Hardly ever the same

21. What appliances have you used for cooking in the past week? You can give multiple answers. Check all applicable options.

Stove
Oven
Airfryer
Fryer
Microwave
Sandwich maker / Toaster
Otherwise:

22. If so, what ingredients did you use when cooking? Also think of spices, butter/oil, sauces, etc.
(open answer)

23. Do you think you can cook well? Mark only one option.
Scale 1-5 I can't cook well to I can cook very well

24. Would you like some help with cooking? Mark only one option.
Yes
No
Maybe
I don't know

25. Do you ever use technology (an app, website, or smart device) to help you cook? Mark only one option.
Yes
no
I do not know

26. If so, what do you use that technology for?
(open answer)

27. Do you wish there was better technology (app, website, smart device) to help you cook? Mark only one option.
Yes
no
I do not know

28. If so, what would that technology do for you?
(open answer)

29. Do you ever go out to eat? Mark only one option.
less than annually
1-5 times a year
6-11 times a year
monthly
2-3 times a month
weekly

2-3 times a week
more than 3 times a week

30. For what reasons do you usually eat out? Multiple answers are correct. Check all applicable options.
to celebrate something
to be with the family
to be with friends
because it's nice
because it's easy
because of tradition
Other:

31. Where do you go when you eat out?
(open answer)

32. Do you ever order food or do you take away food? Mark only one option.
less than annually
1-5 times a year
6-11 times a year
monthly
2-3 times a month
weekly
2-3 times a week
more than 3 times a week

33. For what reasons do you usually order food? You can indicate multiple answers. Check all applicable options.
to celebrate something
to be with the family
to be with friends
because it's nice
because it's easy
because of tradition
Other:

34. What food do you sometimes order (or take away)?
(open answer)

35. What waste do you usually have when preparing the meal? Check all applicable options.
none
washing up
paper
plastic
food scraps
glass
Other:

36. Do you ever throw away food from your stock? Mark only one option.
Never
very little
little
some
a lot

37. Do you ever throw away food leftovers after daily meals? Mark only one option.
Never
very little
little
some
a lot

38. What examples of healthy food technology do you know? You can indicate multiple answers. Check all applicable options.
apps, websites or smart devices that help you prepare meals
apps, websites or smart devices to measure what you take in
apps, websites or smart devices to measure how much food (calories) you consume or need
apps, websites or smart devices that help you do the shopping
apps, websites or smart devices that help you reduce waste or waste

39. Which of the following have you used in the past? You can indicate multiple answers. Check all applicable options.
apps, websites or smart devices that help you prepare meals
apps, websites or smart devices to measure what you take in
apps, websites or smart devices to measure how much food (calories) you consume or need
apps, websites or smart devices that help you do the shopping
apps, websites or smart devices that help you reduce waste or waste
Other:
None

40. In case one of the above is checked, which app, website or smart device did you use for this?
(open answer)

41. Which of the following would you be willing to pay to use them? You can indicate multiple answers. Check all applicable options.
apps, websites or smart devices that help you prepare meals
apps, websites or smart devices to measure what you take in
apps, websites or smart devices to measure how much food (calories) you consume or need
apps, websites or smart devices that help you do the shopping
apps, websites or smart devices that help you reduce waste or waste
Other:
None

42. Have you ever tracked any of the following for the past 3 days? You can indicate multiple answers. Check all applicable options.
What you eat for meals
What you eat in snacks
What you drink
your eating habits
your groceries
your waste
your food preparation habits
your use of technology

43. In case one of the above is checked, how did you do it?
(open answer)

44. Do you have a smartphone? Mark only one option.
Yes
No

45. Do members of your household have a smartphone? Mark only one option.
none
some
all

46. In the case of 'no' and 'none' in answer to the above questions, why don't you have a smartphone?
I think it's unnecessary
I think it's too expensive
I find it awkward
I don't like it
Other:

47. Do you have a smartwatch (a watch or strap that measures your heart rate or temperature, for example)? Mark only one option.
Yes
No

48. Do members of your household have a smartwatch (a watch or band that measures your heart rate or temperature, for example)? Mark only one option.
none
some
all

49. In the case of 'no' and 'none' in answer to the above questions, why don't you have a smartwatch?
I think it's unnecessary
I think it's too expensive

I find it awkward
I don't like it
Other:

50. I can easily provide healthy food.
Scale 1–5 Strongly disagree to Strongly agree

51. I know very well what healthy food is.
Scale 1–5 Strongly disagree to Strongly agree

52. Healthy eating technology is important.
Scale 1–5 Strongly disagree to Strongly agree

53. Healthy eating technology is helpful.
Scale 1–5 Strongly disagree to Strongly agree

54. healthy eating technology takes a lot of time.
Scale 1–5 Strongly disagree to Strongly agree

55. healthy eating technology is unreliable.
Scale 1–5 Strongly disagree to Strongly agree

56. healthy eating technology is easy.
Scale 1–5 Strongly disagree to Strongly agree

57. healthy eating technology is more important to others than to me.
Scale 1–5 Strongly disagree to Strongly agree

58. I find it difficult to eat enough vegetables.
Scale 1–5 Strongly disagree to Strongly agree

59. I find it difficult to eat enough fruit.
Scale 1–5 Strongly disagree to Strongly agree

60. I find it difficult not to eat too much fat.
Scale 1–5 Strongly disagree to Strongly agree

61. I find it hard not to eat too much sugar.
Scale 1–5 Strongly disagree to Strongly agree

62. Members of my household find it difficult to eat vegetables.
Scale 1–5 Strongly disagree to Strongly agree

63. Members of my household find it difficult to eat fruit. strongly agree
Scale 1–5 Strongly disagree to Strongly agree

64. Members of my household find it difficult not to eat too much fat.

Scale 1–5 Strongly disagree to Strongly agree

65. Members of my household find it difficult not to eat too much sugar.
Scale 1–5 Strongly disagree to Strongly agree

66. Healthy food is too expensive.
Scale 1–5 Strongly disagree to Strongly agree

67. Healthy food is tasty.
Scale 1–5 Strongly disagree to Strongly agree

68. Eating healthy is too much work.
Scale 1–5 Strongly disagree to Strongly agree

69. I always have vegetables at home.
Scale 1–5 Strongly disagree to Strongly agree

70. I always have fruit in the house.
Scale 1–5 Strongly disagree to Strongly agree

71. I always have foods with a lot of fat in the house.
Scale 1–5 Strongly disagree to Strongly agree

72. I always have foods with a lot of sugar in the house.
Scale 1–5 Strongly disagree to Strongly agree

73. I would like to use technology to help me cook healthier.
Scale 1–5 Strongly disagree to Strongly agree

74. I would like to use technology to help me eat healthier.
Scale 1–5 Strongly disagree to Strongly agree

75. I would like to use technology to help me drink healthier.
Scale 1–5 Strongly disagree to Strongly agree

76. I would like to use technology to help me have less waste.
Scale 1–5 Strongly disagree to Strongly agree

77. I would like to use technology to help me shop healthier.
Scale 1–5 Strongly disagree to Strongly agree

78. Would you like to know more about the above topics, such as your groceries, preparing meals, your waste and technology? Mark only one option.
Yes
no
I do not know

78. If so, what questions do you have about this?
(open answer)

79. What would you like to change about your groceries, meal preparations and/or waste?
(open answer)
