# Supplementary material for: Low– and Medium–Socioeconomic-Status Group Members’ Perceived Challenges and Solutions for Healthy Nutrition: Qualitative Focus Group Study
Source: JMIR Hum Factors. 2022 Dec 2;9(4):e40123. doi: 10.2196/40123 (PMC9758634; doi:10.2196/40123)
Supplement: Multimedia Appendix 2 [file humanfactors_v9i4e40123_app2.pdf]

Multimedia Appendix II: Overview of Questionnaire Results

Every-day eating practices

In the first questionnaire participants answered questions regarding their regular meals on that day: breakfast, lunch, and dinner. Almost all participants indicated having breakfast (*n* = 40, 95.2%), with bread, dairy, and muesli being the most regularly consumed foodstuffs. 30% drank tea, 35% coffee, 20% water, and another 20% milk. Almost all participants prepared their own breakfast, which was then eaten alone or with partner / family members, at home. Almost all participants (41, 97,6%) also indicated having lunch, with the typical Dutch lunch of sandwiches with diverse toppings and milk being the favourite. Lunch was largely self-prepared (35), eaten at home (31) or at work (10), alone (20), with family or household members (11) or with colleagues (8). All participants had dinner, with the traditional Dutch diet of potatoes, vegetables and meat being most favoured (50.0%), followed by pasta and rice/noodle dishes (28.6%). Dinner was mostly self-prepared (80.9%), with only one participant having food delivered ("because of a special occasion"). 32 participants ate with family or household members, 9 participants ate alone, one ate with colleagues. Most people ate at the dinner table (31.4%) or on the living room sofa (26.2%).

The greater part of participants reported that their consumption pattern on the questionnaire day strongly resembled their habitual eating pattern: 38 of 42 participants said their breakfast was what they always ate; 32 said so about their lunch, and 34 about dinner. The questionnaire also provided insight about other eating habits: 22 participants (52.4%) ate meat or fish every day, and 20 (47.6%) refrained from eating meat or fish at least one day per week. 19 mentioned they were self-described fast eaters (45.2%), 13 said they

saw themselves as slow eaters (31%); participants derived their eating rate from comparing themselves with others ("I am always the first to clean the plate"). When satiated, 4 tended to continue eating (9.5%), 12 sometimes ate too much (28.6%), 4 almost never noticed their satiety and ate what was on their plate and then stopped (9.5%), and 21 stopped eating when they felt full (50%).

34 participants drank coffee regularly, with 15 (35.7) drinking 3–4 cups per day, 10 (23.8%) drinking 5–7 cups per day, and 4 (9.5%) drinking more than 7 cups per day. The majority (26, 61.9%) of the participants drank no alcohol or less than one alcoholic drink per week on average. 10 (23.8%) drank 1–3 drinks per week, and six participants (14.3%) drank more than 3 drinks per week on average. When drinking alcohol, 82.2% drank with partner, friends, or colleagues. 38 of 42 participants went out for dinner sometimes, with 16 (38.1%) doing so 1–5 times per year and 9 (21.4%) 6–11 times per year, 5 (11.9%) at least monthly, and 7 (16.7%) 2–3 times per month. When they did so, their main reasons were celebrating (65.8%), being together with family (50%) or friends (57.9%), because of the taste of the food (50%) or for reasons of convenience (36.8%). 39 participants sometimes ordered take-away food, with 4 (9.5%) doing so once per week, 9 (11.9%) 2 to 3 times per month, 9 (21.4%) once per month, 6 (14.3%) 6–11 times per year, and 15 (35.7%) 1–5 times per year. Main reasons for taking out food were convenience (87.2%) and taste (53.8%).

Self-regulation of snacking and drinking

The second questionnaire asked participants about snacking and drinking behaviours. Twenty (47.6%) participants took a snack in the morning, with cookies and chocolate (45% of reported snacks) the most popular category, followed by fruit (25%) and a typical Dutch snack, Gingerbread ('ontbijtkoek', 25%). Most snacking moments were self-initiated (80% of

snacks) and eaten alone (60%). 29 (69%) participants snacked in the afternoon, mostly between 3PM and 5PM. Again, snacking moments were largely self-initiated (62.1%) and snacks were mostly eaten alone (58.6%). Most popular snack categories were cookies and chocolate (41.1%) and fruit (34.5%). 32 (76.2%) participants snacked in the evening, with cookies and chocolate being the favourite category (50% of snacks), followed by yoghurt (21.9%), fruit (18.8%), and ice cream (12.5%). Evening snacks were often self-initiated (50%), and either eaten together with family or household members (50%) or alone (46.9%).

All participants reported consuming beverages in the morning, with coffee (78.6%), tea (33.3%), water (35.7%) and dairy (11.9%) mentioned most. Only one participant reported refraining from drinking in the afternoon, with coffee (41.5%), tea (34.1%), water (41.5%), dairy (14.6%), lemonades and soft drinks (19.5%), and orange juice (9.8%) mentioned most. In the evening, again only one participant reported no drinks. Those who reported drinks, mentioned coffee (51.2%), tea (26.8%), wine or beer (9.8%), water (39%), lemonades and soft drinks (17.1%) and orange juice (7.3%).

Purchasing, preparing, storing and throwing away food

The third questionnaire asked participants about skills relevant to healthy nutrition: purchasing (healthy) food, preparing food, dealing with waste and leftovers, and using technology for healthy nutrition. Participants usually shopped for groceries 1–2 times per week (50%) or 3–4 times per week (28.6%). Groceries were either purchased by the participant themselves (52.4%) or by the participant and their partner (40.5%), with decisions about what gets bought made by the participants themselves or their partners.

Most participants cooked either all their meals (59.9%) or most of the time (11.9%), if they did not cook, their partner (80%) or children (20%) did the honours. Participants stated to cook the same (kind of) dishes most of the times (26.2%) or at least regularly (50%) and considered themselves quite skilled (42.9%) or at least okay in cooking (42.9%).

After cooking, 97.6% of participants reported having to deal with plastic waste. Food waste (92.9%), food remainders (38.1%), glass containers (40.5%) and paper packaging (35.7%) were other waste categories mentioned. 6 participants (14.3%) never threw away stored food; 69% throw away very little stored food. After a meal, 26.2% never throw out leftover food; 47.6% and 23.8% reported throwing away ‘very little’ and ‘little’ leftovers respectively. Only one participant reported throwing away a lot of stored and leftover food.
